# Characterisation of proteins in excretory/secretory products collected from salmon lice, *Lepeophtheirus salmonis*

**DOI:** 10.1186/s13071-018-2885-6

**Published:** 2018-05-11

**Authors:** Scott Hamilton, Kevin McLean, Sean J. Monaghan, Carol McNair, Neil F. Inglis, Hazel McDonald, Sandra Adams, Randolph Richards, William Roy, Patrick Smith, James Bron, Alasdair J. Nisbet, David Knox

**Affiliations:** 1 Moredun Research Institute, Pentlands Sciences Park, Bush Loan, EH26 0PZ Penicuik, Scotland, UK; 2 Institute of Aquaculture, School of Natural Sciences, University of Stirling, FK9 4LA Stirling, Scotland, UK; 3 Tethys Aquaculture, Ambo, Saffron Waldon, CB11 4JU Essex, England, UK

**Keywords:** *Lepeophtheirus salmonis*, Excretory/secretory (E/S) products, Immunomodulators, Sea lice

## Abstract

**Background:**

The salmon louse, *Lepeophtheirus salmonis*, is an ectoparasitic copepod which feeds on the mucus, skin and blood of salmonid fish species. The parasite can persist on the surface of the fish without any effective control being exerted by the host immune system. Other ectoparasitic invertebrates produce compounds in their saliva, excretions and/or secretions which modulate the host immune responses allowing them to remain on or in the host during development. Similarly, compounds are produced in secretions of *L. salmonis* which are thought to be responsible for immunomodulation of the host responses as well as other aspects of crucial host-parasite interactions.

**Methods:**

In this study we have identified and characterised the proteins in the excretory/secretory (E/S) products of *L. salmonis* using LC-ESI-MS/MS.

**Results:**

In total 187 individual proteins were identified in the E/S collected from adult lice and pre-adult sea lice. Fifty-three proteins, including 13 serine-type endopeptidases, 1 peroxidase and 5 vitellogenin-like proteins were common to both adult and pre-adult E/S products. One hundred and seven proteins were identified in the adult E/S but not in the pre-adult E/S and these included serine and cysteine-type endopeptidases, vitellogenins, sphingomyelinase and calreticulin. A total of 27 proteins were identified in pre-adult E/S products but not in adult E/S.

**Conclusions:**

The assigned functions of these E/S products and the potential roles they play in host-parasite interaction is discussed.

**Electronic supplementary material:**

The online version of this article (10.1186/s13071-018-2885-6) contains supplementary material, which is available to authorized users.

## Background

The sea louse or salmon louse, *Lepeophtheirus salmonis* (Krøyer, 1837) is an ectoparasitic copepod which feeds on the mucus, skin and blood of salmonids [1, 2]. There are eight developmental life stages from planktonic (2 naupliar stages) to infective copepodid through to chalimus (two male chalimus moults and two female chalimus moults), pre-adult (two moults) and adult [3]. Studies of the morphology of *L. salmonis* have revealed tegumental glands and labial (mouthpart) glands which are thought to be involved in secretion [4]. It has been reported that *L. salmonis* produces secretory compounds such as prostanoids and proteolytic enzymes which are involved in host immune-modulation and parasite virulence. The prostanoid prostaglandin E_2_ (PGE_2_) is present in secretory products produced by *L. salmonis* at levels similar to those observed in the saliva of terrestrial arthropods such as ticks [5] and the gene encoding prostaglandin E synthase (LsPGES2) has been identified and characterised in *L. salmonis* [6]. The effects of PGE_2_ have been well documented and these include causing renal vasodilation in rats [7], downregulation of cytokines IFN-γ and IL-2 in mouse T cell lines [8] and reducing adhesion of human lymphocytes to umbilical endothelial cells [9]. Prostaglandin E_2_ has also been shown to downregulate MHC I and II responses in lipopolysaccharide (LPS)-stimulated salmon head kidney cells including isolated leukocytes and cultured SHK-1 cells [10, 11].

Proteases are also secreted by *L. salmonis* [12–14] as well as other parasitic copepod species [15] and are thought to be important for feeding and immune evasion. Serine proteases such as trypsins, thought to be of *L. salmonis* origin, have been reported in salmon mucus [12] and in *L. salmonis* excretory/secretory (E/S) products [11]. Several of these trypsin-like serine proteases have been characterised and have increased mRNA transcript levels in the parasite midgut through all life stages as well as a suggested role in protein digestion [16].

Typically, secretions have been harvested from arthropods using bio-chemical neurotransmitters which induce salivation: tick saliva has been collected successfully following stimulation of salivation by dopamine and pilocarpine [17, 18] and this has led to the identification of numerous proteins using mass spectrometry [19]. Excretory/secretory compounds have also been collected from *L. salmonis* following dopamine stimulation either in solution or after application to the ventral surface of the cephalothorax [5]. The identification of proteins in both somatic and such E/S preparations from *L. salmonis* has been made possible through the generation of genomic and transcriptomic Expressed Sequence Tag (EST) datasets [20, 21]. Transcriptome assembly of RNAseq data from the closely related *Caligus rogercresseyi* has also led to a greater understanding into parasite protein functions and consequently has given insight to host-parasite interactions [22].

The aim of this study was therefore to use these genomic and post-genomic resources to identify and characterize the immunododulatory role of proteins from *L. salmonis* E/S products from two different life stages. Proteins were identified from adult and pre-adult life stages using liquid chromatography-electrospray ionization/tandem mass spectrometry (LC-ESI-MS/MS).

## Methods

### Excretory/secretory material collection

Salmon lice were collected directly from farmed fish at the Atlantic salmon harvesting plant, Mallaig, Scotland and at the Marine Environment Research Laboratory (MERL), Machrihanish, Scotland. Lice were transported directly in sea water maintained at a temperature between 6–8 °C to the Moredun Research Institute, Scotland, where they were maintained at 6 °C in aerated sea water. For the collection of E/S material from adult stages of the parasite, 3 replicate preparations were made from 10 live adult female lice which had been placed into 10 ml of sterile saline (0.85% NaCl) and agitated for 3 h at 6 °C. For pre-adult stages, duplicate preparations of 120 pre-adult lice of mixed sex were placed into 10 ml of sterile saline and agitated for 3 h at 6 °C. Following incubation, the saline solution was harvested and trichloroacetic acid added to it to a final concentration of 10% to precipitate any proteins present. Precipitated material was centrifuged at 10, 000× *g* for 30 min, supernatants were discarded and the pellets allowed to air dry. The pellets were then re-suspended in 50 μl 0.1% Sarkosyl in dH_2_O.

The protein concentrations of each re-suspended pellet were determined using the bicinchoninic acid (BCA) protein assay microplate version according to the manufacturer’s guidelines (Thermo, Peirce, Paisley, Scotland).

### SDS PAGE and LC-ESI-MS/MS

Ten microlitre samples containing between 0.1 and 1.9 mg/ml^-1^ of protein in each E/S preparation were denatured with Lithium Dodecyl Sample buffer (Invitrogen) for 5 min at 100 °C. The proteins were separated by electrophoresis using a 12% Bis-Tris NuPage gel (Invitrogen, Paisley, Scotland) at 150V for 90 min. Following electrophoresis, resolved proteins were visualised with colloidal Coomassie Blue (Simply Blue Safe Stain™, Invitrogen).

Gel lanes were excised and each sliced horizontally from top to bottom to yield a series of 25 equal gel slices of 2.5 mm deep. Each of the resulting gel slices were then subjected to standard in-gel destaining, reduction, alkylation and trypsinolysis procedures [23]. Digests were transferred to low-protein-binding high-performance liquid chromatography (HPLC) sample vials immediately prior to liquid chromatography using electrospray ionisation-tandem mass spectrometry (LC-ESI-MS/MS) analysis. Liquid chromatography was performed using an Ultimate 3000 nano-HPLC system (Dionex, Leeds, England) comprising a WPS-3000 well-plate micro auto sampler, a FLM-3000 flow manager and column compartment, a UVD-3000 UV detector, an LPG-3600 dual-gradient micro-pump and an SRD-3600 solvent rack controlled by Chromeleon™ chromatography software (Dionex). A micro-pump flow rate of 246 μl/min^-1^ was used in combination with a cap-flow splitter cartridge, affording a 1/82 flow split and a final flow rate of 3 μl/min^-1^ through a 5 cm × 200 μm ID (polystyrene-divinylbenzene) monolithic reversed phase column (Dionex) maintained at 50 °C. Samples of 4 μl were applied to the column by direct injection. Peptides were eluted by the application of a 15 min linear gradient from 8–45% solvent B (80% acetonitrile, 0.1% (v/v) formic acid) and directed through a 3 nl UV detector flow cell. LC was interfaced directly with a 3-D high capacity ion trap mass spectrometer (amaZon-ETD, Bruker Daltonics, Bremen, Germany) *via* a low-volume (50 μl/min^-1^ max) stainless steel nebuliser (Agilent, CA95051, United States) and ESI. Parameters for tandem MS analysis were based on those described previously [24].

### Database mining

Deconvoluted MS/MS data in .mgf (Mascot Generic Format) format were imported into ProteinScape™ V3.1 (Bruker Daltonics) proteomics data analysis software for downstream mining of a custom *L. salmonis* database. This custom database was constructed from all the “*Lepeophtheirus salmonis*” protein entries found in the NCBInr database as of January 2018 and comprised 38,092 sequences in total. Database searches were conducted utilising the Mascot™ V2.54.1 (Matrix Science) search engine. Mascot search parameters were set in accordance with published guidelines [25] and to this end, fixed (carbamidomethyl “C”) and variable (oxidation “M” and deamidation “N,Q”) modifications were selected along with peptide (MS) and secondary fragmentation (MS/MS) tolerance values of 0.5 Da whilst allowing for a single 13C isotope. Protein identifications obtained from each of the 25 individual gel slices per lane were compiled using the “protein list compilation” feature within Proteinscape [26]. From the compiled protein lists individual protein identifications were inspected manually and considered significant only if: (i) two peptides were matched for each protein; (ii) peptides were represented by a sequence coverage of > 5%; and (iii) each matched peptide contained an unbroken “*b*” or “*y*” ion series represented by a minimum of four contiguous amino acid residues.

### Functional analysis

To investigate secretory involvement, signal peptide predictions were made from the translated cDNA sequence representing each protein using SignalP prediction software (CBS). Identified proteins were investigated using the Blast2Go software suite [27]. Sequences were blasted against the NCBInr database. Proteins were then assigned into functional groups by searching the InterPro databases and Gene ontology databases. Annotations from both searches were then merged. The Gene Ontology (GO) terms assigned to each protein were then used to construct pie charts based on Biological Process, Cellular Component and Molecular Function. The number of proteins and percentage were included with each GO term. Proteins with no assigned function or associated GO terms were allocated as “Other”.

## Results

### Protein profiles in E/S products

**Fig. 1:**
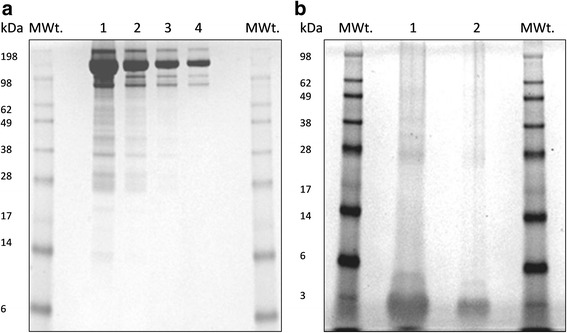
*Lepeophtheirus salmonis* excretory/secretory (E/S) products profiles on Coomassie Blue- stained polyacrylamide gels. **a** Lane MWt: molecular weight standards; Lane 1: undiluted adult female E/S products; Lane 2: 50% dilution of adult female E/S products; Lane 3: 25% dilution of adult female E/S products; Lane 4: 12.5% dilution of adult female E/S products. **b** Lane MWt: molecular weight standards; Lane 1: undiluted pre-adult E/S products; Lane 2: 50% dilution of pre-adult E/S products

In total, 187 individual proteins were identified from E/S collected from adult and pre-adult sea lice. Adult E/S products displayed a wide spectrum of molecular weights with multiple discrete bands present between 24–220 kDa (Fig. 1a). The most dominant proteins with the greatest band intensity ranged between 170–212 kDa. A more diffuse protein gel profile was observed for the pre-adult E/S products with only a few discreet bands seen at 25, 35, 40 and 50 kDa with the dominant band at around 25 kDa (Fig. 1b).

### Proteins common to adult and pre-adult E/S products

Fifty-three proteins were identified as being common to both adult and pre-adult E/S products (Additional file 1: Table S1). Nineteen of these 53 proteins were predicted to have a signal peptide among which were 6 serine type endopeptidases, 2 metalloendopeptidases, 1 peroxidase, 1 hydrolase, 1 hydratase, 1 protein binding, 2 vitellogenin-like proteins and 5 with no assigned function. In total 7 proteins had no assigned function or associated GO terms.

### Adult female E/S products

In total, 107 proteins were identified which were unique to adult lice E/S products (Additional file 2: Table S2). Twenty nine of the 107 proteins were predicted to have a signal peptide and these included 2 serine type endopeptidases, 3 cysteine-type endopeptidases, 3 translocon subunits, 3 vitellogenins, 4 isomerases, 1 transferase, 1 hydrolase, 3 calcium binding proteins, 1 transmembrane emp24 protein 1 lysozyme, 1 sodium/calcium exchanger, 1 fibronectin, 1 heat-shock protein, 1 transforming growth factor, 1 endoplasmin and 2 uncharacterised proteins. The high molecular weight dominant bands observed in Fig. 1a correlate to the egg derived vitellogenin proteins (vitellogenin 1 and 2 and vitellogenin-like). The proteins in this group show the highest Proteinscape scores and sequence coverage and are represented within each replicated sample. Five proteins had no assigned function or associated GO terms.

### Pre-adult E/S products

A total of 27 proteins were identified in pre-adult E/S products but not in adult E/S. Eight of the 27 proteins were predicted to have a signal peptide and these included 3 serine type endopeptidases, 2 cysteine type endopeptidases, 1 phosphatase, 1 acetylcholine receptor and 1 with no assigned function. (Additional file 3: Table S3). In total, 4 proteins had no assigned function or associated GO terms.

### Assigned function of E/S products

**Fig. 2:**
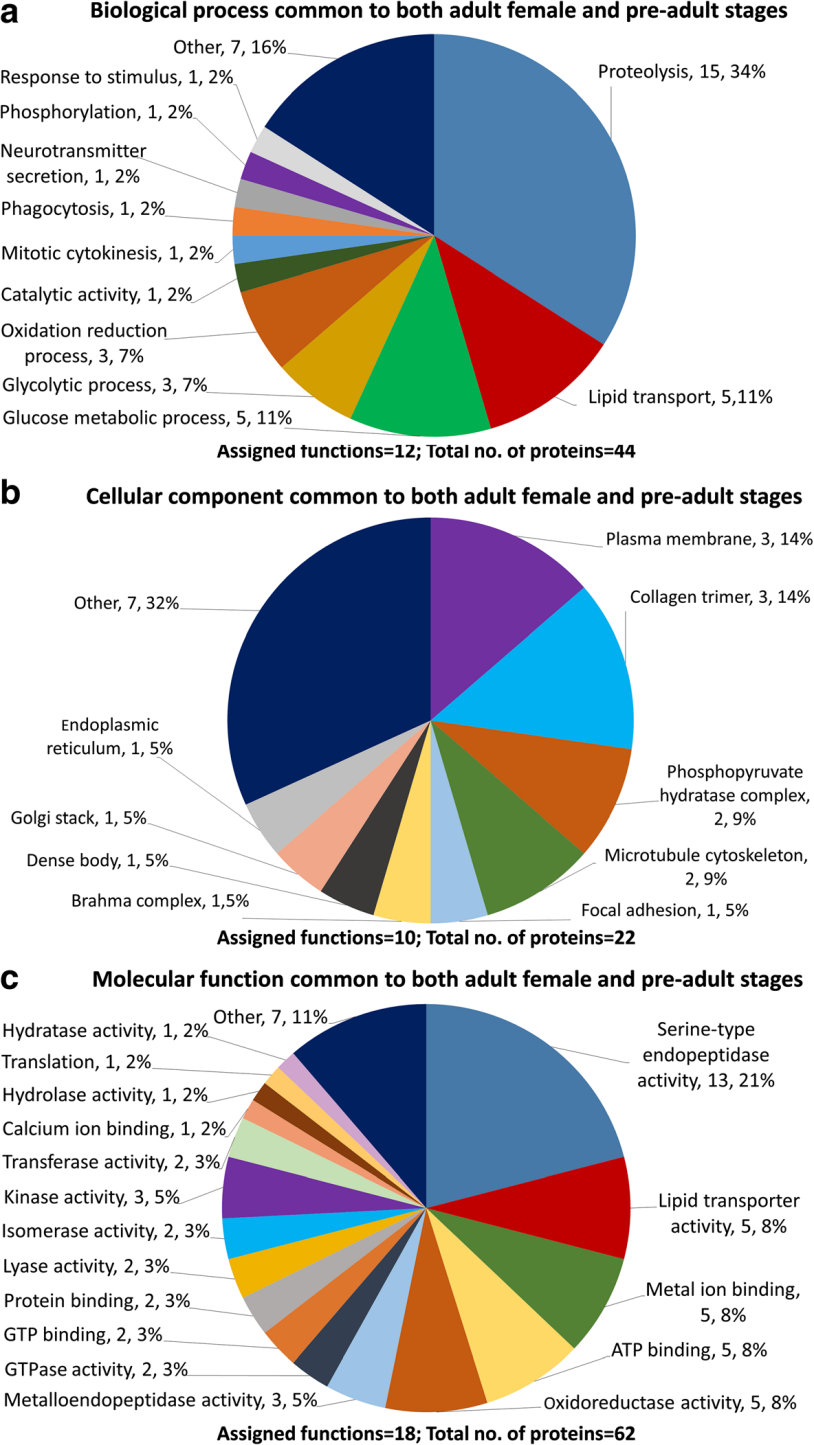
*Lepeophtheirus salmonis* excretory/secretory (E/S) products common to both adult female and pre-adult stages. **a** Biological Process. **b** Cellular Component. **c** Molecular Function

**Fig. 3:**
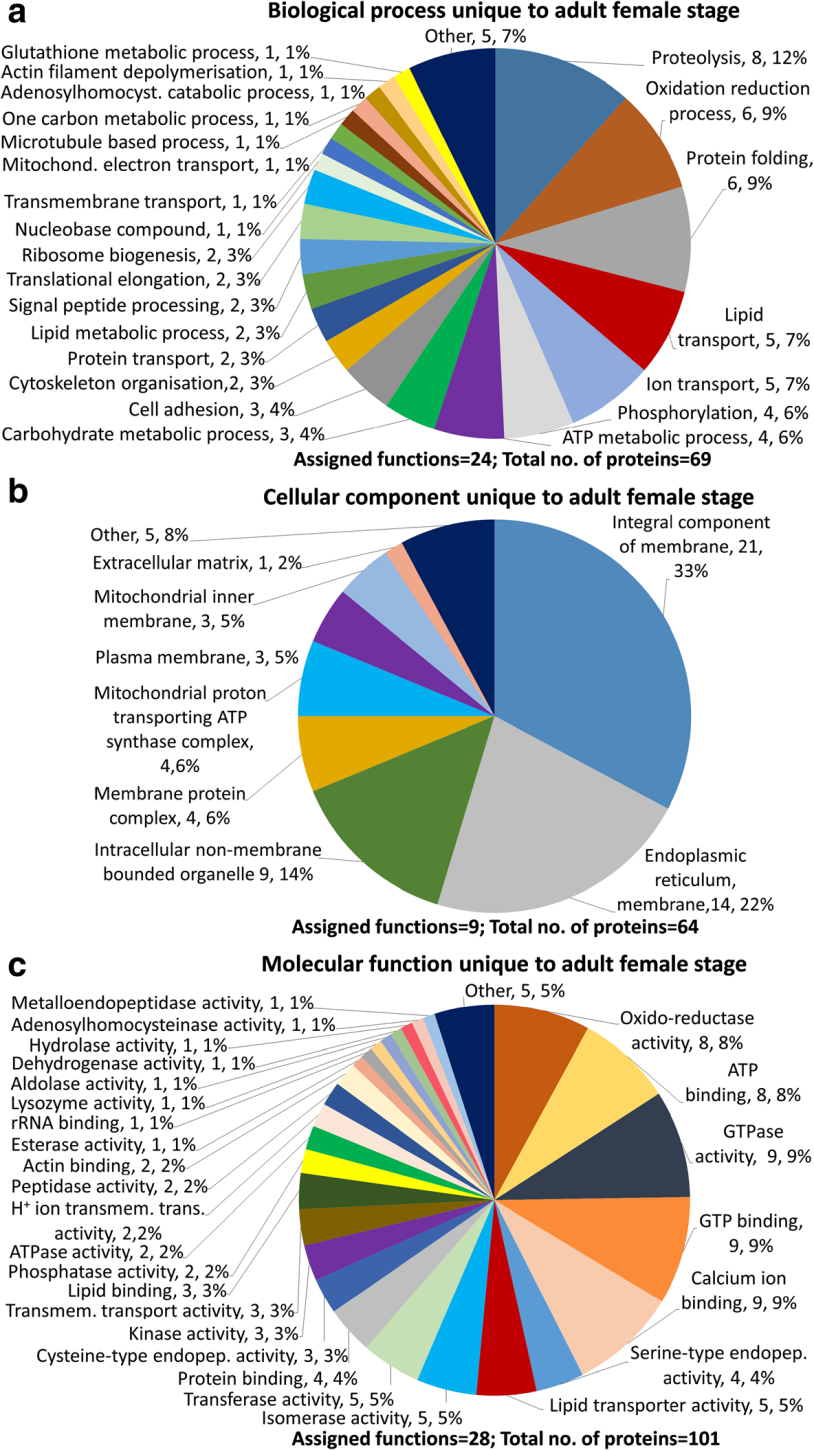
*Lepeophtheirus salmonis* excretory/secretory (E/S) products unique to adult female stage. **a** Biological Process. **b** Cellular Component. **c** Molecular Function

**Fig. 4:**
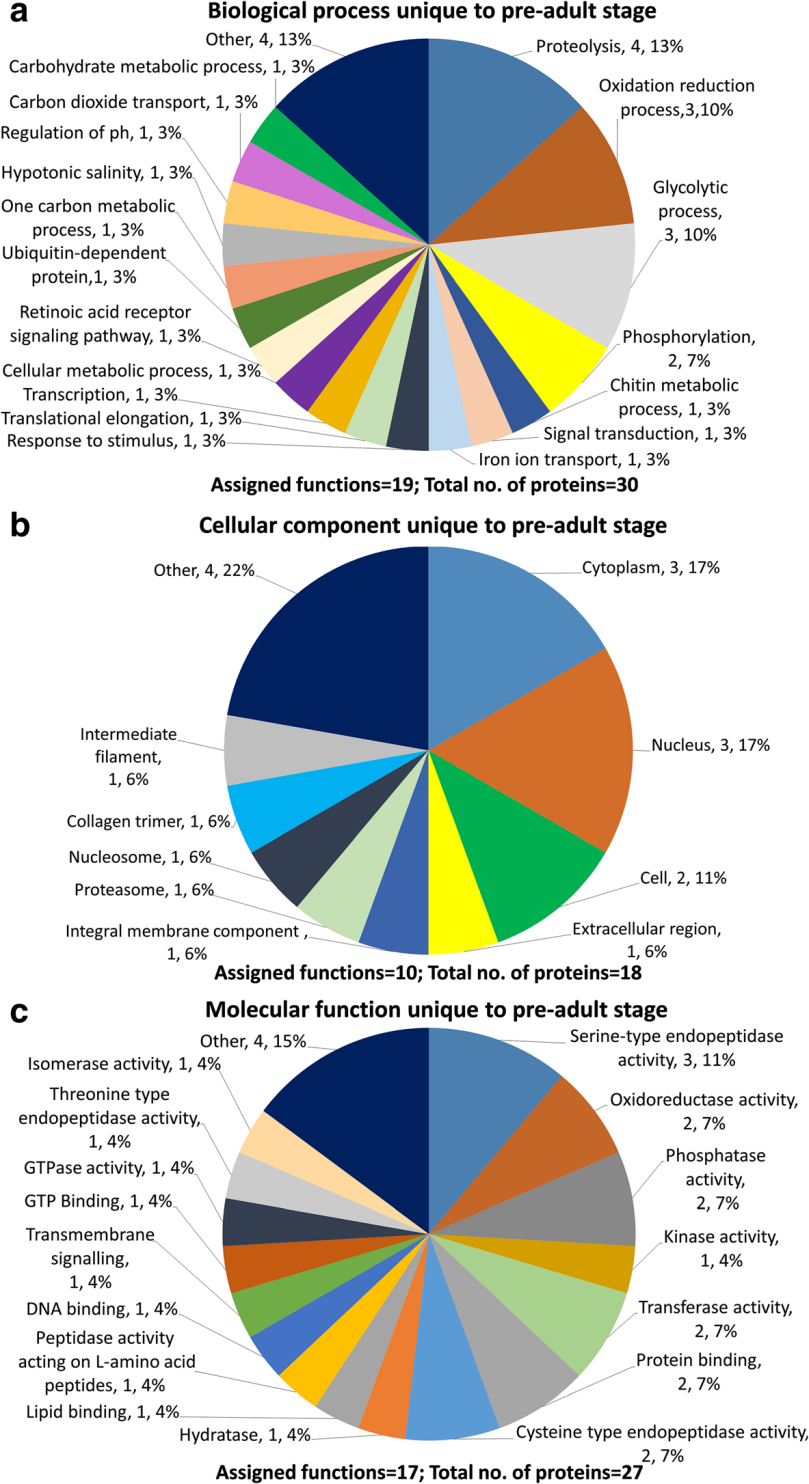
*Lepeophtheirus salmonis* excretory/secretory (E/S) products unique to pre-adult stage. **a** Biological Process. **b** Cellular Component. **c** Molecular Function

Proteins common to the E/S products of both life stages (Fig. 2) or those that were detected only in adult stage (Fig. 3) or only in pre-adult stage (Fig. 4) were assigned to Biological Process, Cellular Component and Molecular Function. Proteins associated with proteolysis accounted for 34% of biological processes common to both life stages (Fig. 2a), 12% in adults only (Fig. 3a) and 13% in pre-adults only (Fig. 4a). Proteins involved in lipid transport were observed common to both stages at 11%, all being vitellogenin-like proteins and in adults only at 7% including vitellogenin 1, vitellogenin 2, vitellogenin-like and microsomal triglyceride transfer protein. No lipid transport proteins were observed only in pre-adults. Metabolic processes such as carbohydrate metabolism accounted for most proteins common to both stages, adult only and pre-adult only. Chitin metabolism was present only in pre-adults.

Proteins assigned to have cellular component consisted mostly of membrane proteins common to both life stages (Fig. 2b), adults only (Fig. 3b), and pre-adults only (Fig. 4b). A large proportion of proteins were assigned as other and had no assigned function or associated GO terms.

Proteins assigned to have molecular function, presented a wide range of enzymes observed in both life stages including metallopeptidases, reductases, transferases, kinases, GTPases, isomerases, lyases, hydrolases and hydratases. Serine type endopeptidases common to both stages, adult only and pre-adult only, constituted 21%, 4% and 11%, respectively (Figs. 2c, 3c, 4c). Cysteine endopeptidases were observed in adult only and pre-adult only stages at 3% and 7%, respectively. One threonine peptidase was detected in pre-adult E/S products only. Proteins associated with oxidoreductase activity were observed in 8%, 8% and 7% common to both stages, adult only and pre-adult only, respectively. Enzymes unique to adult only E/S products were dehydrogenase, aldolase, esterase, lysozyme and adenosylhomocysteinase. Protein, GTP, DNA, calcium and ion-binding proteins were among those proteins common to both stages, in adult and pre-adult only samples.

## Discussion

Proteins secreted by salmon lice have long been thought to be important for the success of the parasite in evading the host immune response [12, 28]. Here, we have identified a suite of proteins excreted or secreted by adult and pre-adult salmon lice *L. salmonis* which may aid in understanding the interactions between the salmon louse and its host *S. salar*. In the present study, 21% of the 62 proteins, assigned to molecular function that we identified as being common to adult and pre-adult lice were serine peptidases, specifically trypsins and 5% were metallopeptidases. In addition, a serine collagenase was represented amongst these “common to both stages” proteinases which potentially facilitate feeding and digestion of host tissues, specifically for the breakdown of connective tissue and in this case mucus, skin and blood digestion. Metallopeptidases have been identified in the saliva of ticks (*Ixodes scapularis*) where they have effects on the ability of the tick to transmit pathogens by reducing adherence of polymorphonuclear leukocytes to the spirochete *Borrelia burgdorferi*, the causative agent of Lyme’s disease [29]. Other potential immunomodulatory proteins were the anti-oxidant family proteins, thioredoxin 2 and thiol peroxiredoxin isoform X3. These proteins may play a role in immune modulation by protecting against host reactive oxygen species as has been shown in helminth parasites [30]. Salmon louse E/S products have previously been shown to have immunomodulatory properties, for example causing decreased expression of IL-1β and MHC I in salmon head kidney macrophages [5].

Arginine kinase, which is solely expressed in invertebrates, is also present in *L. salmonis* (Additional file 1: Table S1) and in ectoparasite species, and is thought to play a key role in energy metabolism facilitated by buffering levels of intracellular adenosine triphosphate in muscle tissue [31]. Arginine kinase is a significant shellfish allergen [32] and has recently also been identified as an important allergen of house dust mites [33].

The hydratase protein, ganglioside GM2 activator-like protein observed in both life stages, contains an ML domain homologous to that found in Der p2 which is a major allergen in the house dust mite *Dermatophagoides pteronyssinus* [34]. In the hard tick *Ixodes ricinus*, the expression of the transcript encoding a Der p2 homologue is substantially increased during feeding and the encoded protein has been shown to have potential immunomodulatory properties on the host [35].

The processes involved in collecting and analysing E/S proteins, such as live lice collection and culture, genomic resource for the identification of specific peptides and bioinformatics to understand structure and function make the type of study reported here technically challenging. Fast et al. [11] reported low secreted protein yields from mixed life stage, dopamine induced *L. salmonis* with mostly low molecular weight proteins observed by SDS-PAGE. In our study presented here, the protein band profiles of E/S products observed from adult lice and pre-adult lice were markedly different. Protein gel profiles of pre-adult E/S products, show a very discreet group of bands between 20–55 kDa in size amongst which 11% of the identifiable proteins were serine type endopeptidases. Serine enodpeptidases, specifically trypsins, from cattle warble fly (*Hypoderma lineatum*) larvae have been associated with complement C3 degradation in cattle [36], demonstrating a possible immunomodulatory role for these molecules. Experiments carried out by Firth et al. [12] suggest that serine endopeptidases are secreted by *L. salmonis* in the presence of salmon mucus and, given the relatively high number of serine peptidases identified here in the pre-adult stages of the parasite, these proteins may be of key importance to the success of the louse in evading the host immune system before going into the final stage in the parasite life-cycle.

In contrast, the adult lice E/S products contained a wide spectrum of proteins from 20–220 kDa in size. Accordingly, the protein yield was higher with a broader diversity of protein types identified. This was despite using much greater numbers of pre-adult lice (*n* = 120) for collection of secretions than adult lice (*n* = 10). The differential protein profile between stages may reflect the importance of these proteins for feeding. On becoming sexually mature, adult lice appear to feed on blood more readily than pre-adult lice [1, 2]. In our study, we identified 3 cysteine proteases including Cathepsin L suggesting an important facilitatory role in increased feeding in this stage. A Cathepsin L has been shown to be vital in blood meal digestion in hard ticks (*I. ricinus*) [37] and a *Fasciola hepatica* cathepsin L has also been shown to have immunomodulatory properties by suppressing ovine T-lymphocyte proliferation and CD4 expression during parasitism by liver flukes [38].

It is worth noting that in previous studies, prostaglandins, thought to be important immune modulators, were observed in both excretory/secretory and secretory only sample collections [5]. However, prostaglandin synthase E_2_, although observed here, did not meet stipulated confidence criteria sufficiently to merit inclusion in the catalogue of identified ES proteins. Eichner et al. [6] recently reported that knockdown of the gene encoding PGE_2_ synthase by RNAi did not adversely affect parasite survival or the ability to infect the host and that the encoded protein was most likely involved in muscle contraction in planktonic stages and reproduction in adult females. Thus, the role of PGE_2_ in salmon louse immune-modulatory mechanisms remains to be determined.

## Conclusions

The proteins identified in this study give an insight into the complexity of *L. salmonis* secretions, from serine proteases and redoxins which may be involved in host immune evasion, to metallopeptidases and cysteine peptidases such as cathepsin L thought to be involved in feeding. Utilizing the most up to date genomic resources currently available for *L. salmonis,* we can confidently identify a significant portion of the entire louse E/S protein complement. The biological functions of the E/S proteins identified here facilitate a better understanding of the ways in which the sea louse parasite interacts with its host environment.

## Additional files

Additional file 1: **Table S1.** E/S products common to both adult female and pre-adult stages. Data includes accession number, protein name, Proteinscape scores, no. of matched peptides, sequence coverage, signal peptide prediction and peptide sequence. (XLSX 32 kb)

Additional file 2: **Table S2.** E/S products unique to adult female stage. Data includes accession number, protein name, Proteinscape scores, no. of matched peptides, sequence coverage, signal peptide prediction and peptide sequence. (XLSX 48 kb)

Additional file 3: **Table S3.** E/S products unique to pre-adult stage. Data includes accession number, protein name, Proteinscape scores, no. of peptides matched, sequence coverage, signal peptide prediction and peptide sequence. (XLSX 18 kb)

## Abbreviations

E/S: excretory/secretory
LPS: lipopolysaccharide
LC-ESI-MS/MS: liquid chromatography-electrospray ionisation-tandem mass spectrometry
MERL: Marine Environment Research Laboratory
NaCl: Sodium chloride
NCIBnr: National Centre for Biotechnology Information non-redundant
HPLC: high-performance liquid chromatography
GO: Gene Ontology
BCA: bicinchoninic acid
kDa: kilodalton
EST: expressed sequence tag
SDS-PAGE: sodium dodecyl sulfate polyacrylamide gel electrophoresis
PGE_2_: prostaglandin synthase E_2_
MHC: major histocompatibility complex
IFN- γ: interferon gamma
IL-2: interleukin 2
GTP: guanidine triphosphate
RNAseq: RNA sequencing
RNAi: RNA interference
